# Comparative effectiveness of COVID-19 vaccines among health students, focusing on methodological concerns

**DOI:** 10.1017/ash.2026.10338

**Published:** 2026-04-07

**Authors:** Banu Cakir, Ahmet Sertcelik, Eda Karadogan, Hanife Uzar, Mithat Temizer, Seyma Aliye Kara, Mustafa Enes Ozden, Bilal Buzgan, Damla Ozyurek, Alpaslan Alp, Asli Pinar, Duygu Ayhan Baser, Hilal Aksoy, Izzet Fidanci, Nursel Calik Basaran, Sehnaz Ozyavuz Alp, Volkan Arslan, Burcin Sener

**Affiliations:** 1 Hacettepe Universitesi Tip Fakultesi, Türkiye; 2 Ankara Yıldırım Beyazıt Üniversitesi Tıp Fakültesi: Ankara Yildirim Beyazit Univ, Türkiye; 3 Ministry of Health, Türkiye, Antalya, Türkiye; 4 Republic of Turkey Ministry of Health: Turkiye Cumhuriyeti Saglik Bakanligi, Türkiye; 5 Manisa Celal Bayar University: Manisa Celal Bayar Universitesi, Türkiye; 6 Hacettepe University: Hacettepe Universitesi, Türkiye; 7 Bandirma Onyedi Eylul Universitesi, Türkiye

## Abstract

**Introduction:**

With emerging variants of SARS-CoV-2, there is an increasing demand for comprehensive data on vaccine effectiveness disaggregated by vaccine type and/or country to frame future pandemic readiness plans.

**Design, setting, participants:**

We investigated comparative effectiveness (VE) of CoronaVac® and Comirnaty® vaccines among health students, considering potential risk factors. An open, prospective cohort study was conducted to follow participants with valid COVID-19 vaccinations, up to 2 years. Investigations included VE against symptomatic PCR-confirmed COVID-19 infections, along with vaccine-induced humoral immunity (including durability) and potential methodologic threats to conclusive decisions.

**Results:**

Symptomatic COVID-19 incidence rate was 4.24 (95% CI = 3.69–4.86) per 10,000 person-days among 1133 students (46.6% males) over 478,466 person-days. Taking a primary series/booster with CoronaVac as the reference, a primary series with Comirnaty or a Comirnaty booster protected students up to twenty times early in the pandemic, adjusting for covariates; significance disappeared in the Omicron period, though. Unexpected upsurges in virus-specific antibody levels, starting 3-6 months after the last vaccination when titers decreased to almost nill, suggested that disproportionality in vaccine durability could have led to a bias towards the null in VE estimates, due to mediator role of undetected breakthrough infections.

**Conclusion:**

Hybrid immunity may differentially deplete the susceptibles in either arm of the study, leading to bias in VE estimations. High infection rate in Omicron period might have augmented this bias, favoring the protective effect of the less potent vaccine. Periodic PCR testing as an integrated measure in future VE studies can avoid such bias.

## Introduction

Coronavirus Disease 2019 (COVID-19) spread across the globe approximately 3.5 months after its first identification and was declared as a pandemic early in 2020.^1^ In Türkiye, the first case was officially announced on March 10, 2020.^2^ Health care workers and those aged 65 years or older were prioritized in primary vaccination against COVID-19, starting from January 14, 2021. By that date, official number of SARS-CoV-2 infections in Türkiye had reached 2,412,505 residents, with 24,640 COVID-19-related deaths. In less than a year, 85.7% of all residents were vaccinated against COVID-19 with a least 2 doses (n = 53,195,248).^3–4^

Health students were at high risk of exposure given their long working hours and/or night shifts, low experience of self-protection against infected patients, and apparent neglect to full compliance with non-pharmacological preventive measures. Investigation of infection rates among these students was also valuable because they could also transmit infection within the hospital due to rapid turnover across different departments during their rotations and close contact with other students in dormitories, student lounges, cafeteria, and so. Unfortunately, this special group had been left behind in local and global health workers-related COVID-19 research.^5^

We focused our study on senior students of dental and medical schools, with 3 major objectives: (1) to investigate comparative effectiveness and durability of two different COVID-19 vaccines, based on symptomatic event-based surveillance over 2 consecutive years; (2) to periodically study the level of anti-SARS-CoV-2 spike-RBP IgG antibodies by vaccine type/dosages/time through the end of follow-up; and (3) to scan for factors that might bias the comparative analysis for vaccine effectiveness (if any).^6^

## Materials and methods

### Study location, participants, and design

The Hacettepe University Coronavirus Vaccinateds’ Health Cohort-Students of Health Sciences (HU-CoVaCS) study included senior students medical and dental faculties of Hacettepe University, who were actively working in outpatient clinics and hospital wards over the pandemic, including the full-closure periods. Hacettepe University (HU), founded in Ankara in 1967, owns 3 hospitals in the same campus.^7^

**Figure 1.**
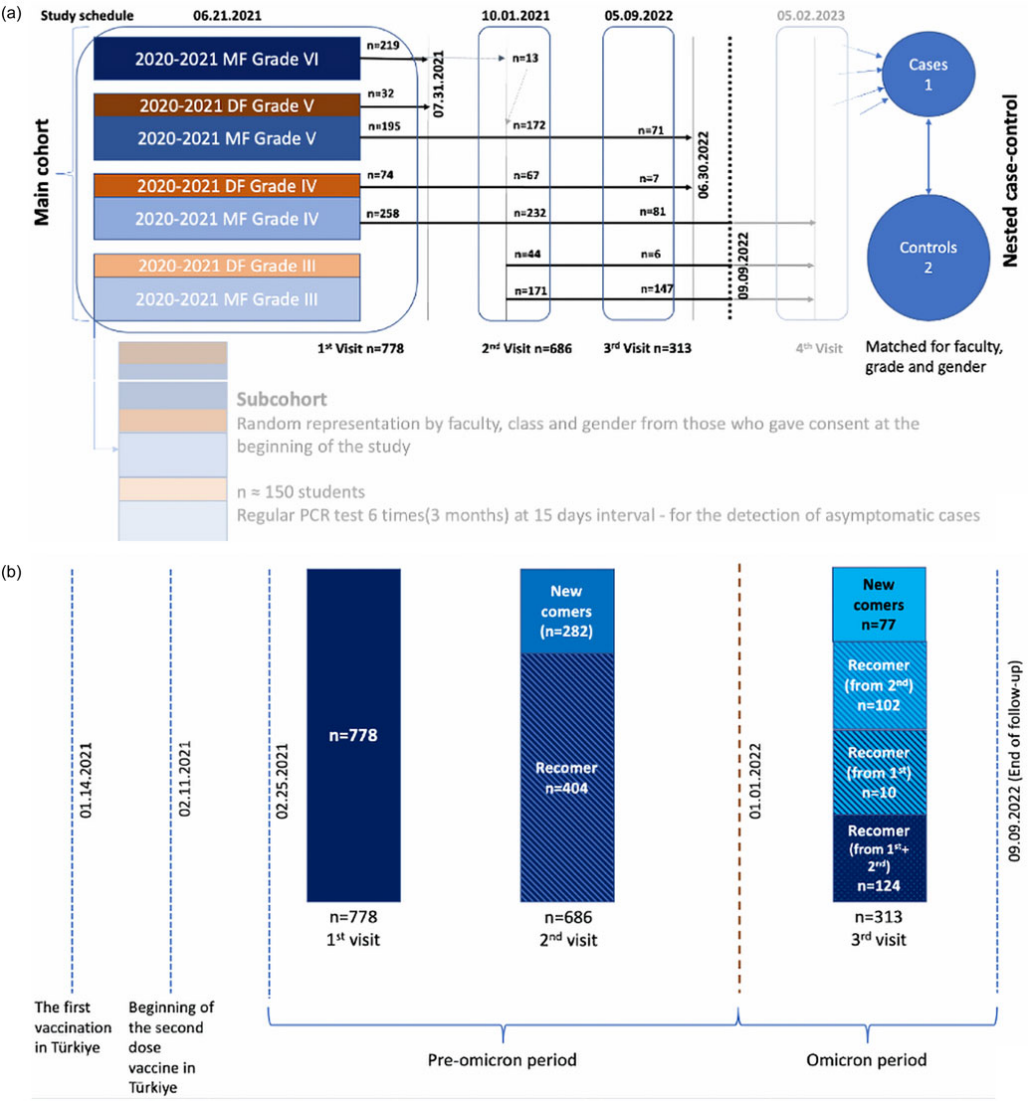
Design and flow of the study (first 18 months of the cohort).

HU-CoVaCS was designed as a prospective, open cohort of 2.5 years, with 4 successive visits, 3-to-12 months apart (Figure 1). Besides a main cohort of health and COVID-19-related investigations, a (school-, grade-, and sex-) matched, nested (1:2) case-control study and a subcohort (for periodic PCR testing) were integrated into the main cohort. All students of grades 4, 5 and medical interns were invited for the study and those who participated in any of the 4 visits were included in the study cohort, with no exclusion criterion. Study participants were followed through September 9, 2023, graduation, or when they wanted to discontinue, whichever came the first. This manuscript is based on the first 3 visits of the main cohort; presented analyses are restricted to COVID-19-related examinations and lab tests, only. Details of the study protocol can be reached for further inquiries.^6^

#### Ethics approval and consent to participate

This study was performed in line with the principles of the Declaration of Helsinki. Approval was granted by the Hacettepe University Clinical Researches Ethics Committee (Approval date: February 23, 2021, number: KA-21034). Written informed consent to participate was obtained from all participants included in the study.

#### Data sources, measurements, and definitions

Each visit started with an online self-administered questionnaire, anthropometric measurements, and a full physical exam. Blood samples were drawn by registered nurses; anti-SARS-CoV-2 quantitative IgG antibody measurement was performed using the anti-SARS-COV-2 spike- RBP IgG kit (Architect, Abbott Laboratories, IL, USA) in hospital’s central laboratory. Results ranged between 0 and 40,000 in antibody unit (AU)/mL; values <50 were considered as negative. The AU/mL value obtained was multiplied by 0.142 to calculate binding antibody unit (BAU)/mL, corresponding to the World Health Organization (WHO) standard.^8^ The Artus SARS-CoV-2 Prep&Amp UM real-time PCR kit (Qiagen, Germany) was used to detect SARS-CoV-2 in clinical samples and positive test results were monitored daily.

Students with any COVID-19 symptom could either admit to HU Hospitals for SARS-CoV-2 real-time polymerase chain reaction (RT-PCR) or have it done in any other hospital for free, as was true for any Turkish citizen. Incident COVID-19 cases were detected daily from the HU Electronic Health Record System. PCR test results conducted outside the hospital and vaccination-related information were verified based on students’ e-Nabız records, including the period before the first visit in June 2021. e-Nabız is the personal electronic health record of Turkish citizens, with a national coverage of 82%.^9^ The system was equipped with new services in the pandemic to integrate information on COVID-19-related tests, hospitalizations, and vaccine-related information. Each participant had secure access to her/his e-Nabız data and personally confirmed the dates they were requested for the study.

Two vaccines in use for national COVID-19 immunization program were studied, namely the inactivated whole virus vaccine CoronaVac (Sinovac Biotech, Beijing, China) and the m-RNA vaccine Comirnaty (BioNTech SE, Mainz, Germany); both vaccines were against the wild-type virus.

Vaccination status was grouped as: those with a primary vaccination scheme with CoronaVac (CC) (2 doses) or Comirnaty (BB) (2 doses); those with a primary vaccination scheme with CoronaVac and reminder doses with CoronaVac were classified as CoronaVac homologous (CC homologous booster), and those with reminder doses with Comirnaty were classified as CoronaVac heterologous (CC heterologous booster); lastly, those with a primary vaccination scheme with Comirnaty and reminder doses with Comirnaty were classified as Comirnaty homologous (BB homologous booster). Those with incomplete primary vaccination schemes were classified as incomplete vaccination and a few whose vaccination scheme did not comply with any of these conditions were classified as “miscellaneous.” Throughout the text, vaccination status refers to “valid” vaccination, that is 14 days following at least 2 doses of (any) COVID-19.

For analysis, vaccination-based immunity follow-up was initiated 14 days after the completion of the primary schedule (ie, two doses of vaccine, at least 28 d apart), and total follow-up was extended through the date of symptomatic COVID-19 or the last study visit attended (July 31, 2021, June 30, 2022, or September 09, 2022) (Figure 1). Graduates were censored at the time of graduation. We stratified analyses into pre-Omicron and omicron periods, with a cutoff date of January 01, 2022; variations in the size of population at risk was presented in Figure 1.

### Statistical analysis

Descriptive statistics included numbers and percentages for categorical data; mean, standard deviation and median (25–75 percentiles or interquartile range: IQR) for continuous data. Incidence rate, cumulative incidence, and corresponding 95% confidence intervals (CIs) were calculated.^10^ Multiple linear regression was performed to model antibody levels by vaccine type/scheme (as the main exposure). Time since valid vaccination, age, gender, faculty, grade, adherence to non-pharmacologic measures, total number of comorbidities, and RT-PCR-confirmed history of COVID-19 (if any) were examined as covariates in models. The association between anti-SARS-CoV2 antibodies and case status was investigated based on the most recent antibody titer, that is obtained prior to becoming a case, or the time of censoring (for non-cases)—to control for multiple measurements. Kaplan-Meier cumulative hazard curves for test-confirmed symptomatic COVID-19 were depicted for pre-Omicron and Omicron periods to determine protective effects of vaccine types; Log-rank test was used for significance. Cox proportional hazard models were constructed separately for Omicron period, and the preceding period: The first (core) model included the vaccination scheme as the main exposure; the second model additionally included variables on nonpharmacologic preventive measures. The final model included core model variables plus age, gender, faculty, grade, and presence of comorbidity: BMI and smoking status were excluded from the full model due to poor fit.

Analyses were performed with IBM SPSS v23 (Armonk, New York, U.S.A). Type 1 error was set at 0.05. The epidemic curve was plotted with Microsoft Excel (Microsoft Corporation, Redmond, Washington, U.S.A). The scatter plot and curve of antibody levels according to vaccine types were made with R (The R Foundation for Statistical Computing) version 4.2.2 software and “*ggplot*” package.

## Results

A total of 1,133 health students (46.6% males) participated in at least one of the three subsequent visits over the first 18 months of HU-CoVaCS; each visit was completed in 10 days, with initiation dates as marked on Figure 1. From February 25, 2021, a total of 203 symptomatic COVID-19 cases were detected through September 9, 2022. The cumulative incidence of PCR-confirmed symptomatic COVID-19 infection was calculated as 17.9% in 18 months, with a corresponding COVID-19 incidence rate of 4.24 (95% CI = 3.69–4.86) per 10 000 person-days. Similarities in distribution of HU-CoVaCS cases and those in general population over time was remarkable (Suppl. Figure 1).^11^

Distribution of the sociodemographic, health-related, and COVID-19-related characteristics of the participants including adherence to non-pharmacologic preventive measures (either in hospital or in social life) during 30 days preceding the survey were presented in Table 1. The mean age of our study participants was 23.5 years, with a standard deviation of 1.9 years. The median BMI was 22.9 kg/m^2^ (25%–75% = 20.5–25.3); prevalence of overweight/obese students was 27.5% of participants. Of the participants, 1,117 were followed for a total of 424,528 person-days, after valid vaccination. The mean follow-up was 425.34 ± 164.28 person-days and the median was 490.0 (25%–75% = 320.0–561.0) person-days. Table 2 presents the distribution of potential risk factors for COVID-19 at the time of the most recent cohort visit the student participated in, preceding the COVID-19 case incident or the time of censoring, whichever came the first.^6^

**Table 1.** Distribution of sociodemographic, health- and COVID-19-related characteristics of study participants

Characteristics	Number	Percent
Male gender	528	46.6
**Faculty**		
Medicine	959	84.7
Dentistry	173	15.3
**Grade**		
IV	367	32.4
V	379	33.5
VI (medical interns, only)	386	34.1
**Smoking status**		
Current smoker	222	23.4
Never smoked/Ex-smoker	725	76.6
**Adherence to all three non-pharmacologic public measures** ^a^ **inside the hospital**		
Always/usually	1,035	94.6
Rarely/never	59	5.4
**Adherence to all three non-pharmacologic public measures** ^a^ **in public places**		
Always/usually	764	71.3
Rarely/never	307	28.7
**Participation in crowded settings over the preceding 3 months**		
Always/usually	186	20.2
Rarely/never	736	79.8

a Adherence to masking-distancing-personal hygiene was inquired as “all”.

**Table 2.** Distribution of COVID-19-related potential risk factors and corresponding antibody titers at the most recent visit based on case status

Variable of Interest	Mean ± Std. Dev.^a^	Median (25%–75%)
Age (years) (n = 1,133)	23.5 ± 1.9	23.0 (22.0–24.1)
Body mass index (kg/m^2^) (n = 1,069)	24.0 ± 20.8	22.9 (20.5–25.3)
Underweight (<18.5)	76	7.1
Normal (18.5–24.9)	698	65.4
Overweight (25.0–29.9)	227	21.3
Obesity (≥30.0)	66	6.2
Total comorbidity count (n = 1,133)	.3 ± .6	0 (0–0)

a Std. dev., standard deviation.

The majority of COVID-19 vaccine scheme administered was CoronaVac as the primary series, followed with a Comirnaty booster; referred to as CC-with heterologous booster (69.9%) (Suppl. Table 1). Three students with incomplete primary series, one student with a TurkoVac© booster following primary series with Comirnaty and three students with one dose of CoronaVac with various numbers of Comirnaty doses afterward were deleted from modeling, for simplicity.

Given the prominent individual variation among vaccination dates and multiple antibody measurements, association between antibody titers and symptomatic infection was studied using the most recent cohort visit preceding the case status/censoring time (Suppl. Table 2); follow-up time was calculated similarly. The mean anti-SARS-CoV-2 lgG antibody titer was 1729.3 BAU/ml, with a standard deviation of 1780.1; the median was 1,126.4 (25%–75% = 165.1–2,677.8) BAU/ml.

The multiple linear regression model (Suppl. Table 3) for antibody titer also revealed significant associations with gender, faculty type, and type of COVID-19 vaccine used, adjusting for age (*P* = .13); adherence to non-pharmacological measures in hospital (*P* = .81) or in public places (*P* = .97); participation in crowded places (*P* = .21); history of RT-PCR-confirmed symptomatic COVID-19 infection (*P* = .30); presence of comorbidity (*P* = .40). In this model, considering CC as the reference group, time since the last valid vaccination was negatively associated with antibody titers and having a booster dose with Comirnaty was associated with high antibody levels, regardless of the primary series.

**Figure 2.**
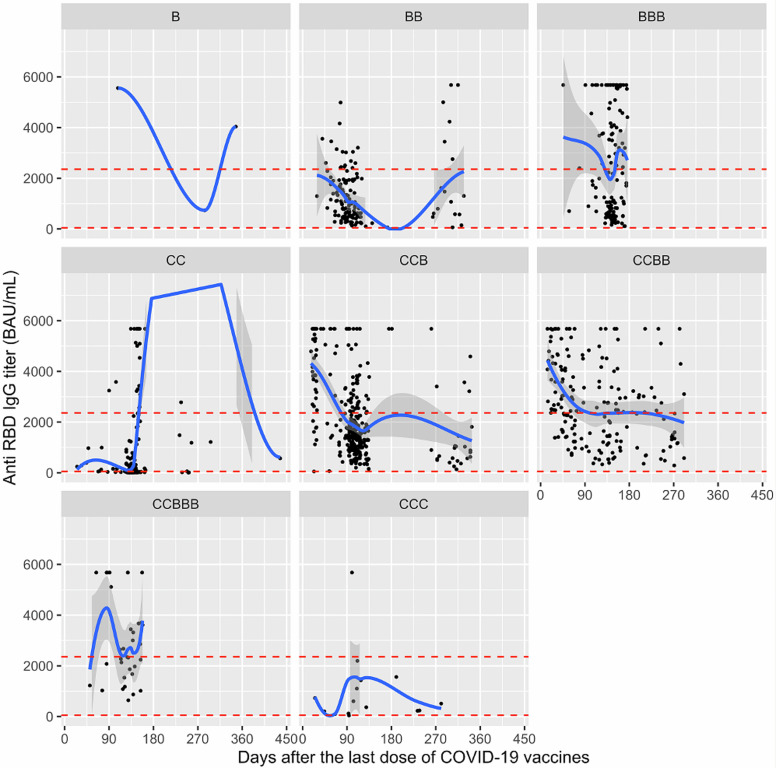
Distribution of anti-SARS-COV-2 spike- RBP igG antibodies among students with different vaccination schemes (for all vaccinations, antibody titer measured after at least 14 days following the last vaccine dose/booster are presented). (a) daily COVID-19 cases, (b) daily cases for HU-CoVaCS compared to corresponding cumulative case numbers in Türkiye over corresponding weeks (based on data from WHO) B: Comirnaty (one dose) (n = 3), BB: Comirnaty-2 doses (n = 136), BBB: Comirnaty as primary series with a Comirnaty booster (n = 119); CC: CoronaVac-2 doses (n = 328), CCB: CoronaVac as primary series with a Comirnaty booster (n = 299), CCBB: CoronaVac as primary series with 2 Comirnaty boosters (n = 178), CCBBB: CoronaVac as primary series with 3 Comirnaty boosters (n = 34), CCC: CoronaVac as primary series with a CoronaVac booster (n = 16).

A remarkable decline in antibody titers was observed every 3 months following vaccination (Figure 2). Scatter graphs were used to describe the level of the most recent anti-SARS-COV-2 spike RBP IgG antibody titers (in BAU/ml) since the last valid vaccination. Based on the initial national COVID-19 vaccination scheme of a primary series of CoronaVac and late introduction of Comirnaty into the vaccination scheme, the longest follow-up (437 d) was for those with CC and antibody levels decreased as time passed. Unexpectedly, surges in antibody levels 90-to-180 days after the last vaccine dose (without any detected infection) were remarkably high.

**Figure 3.**
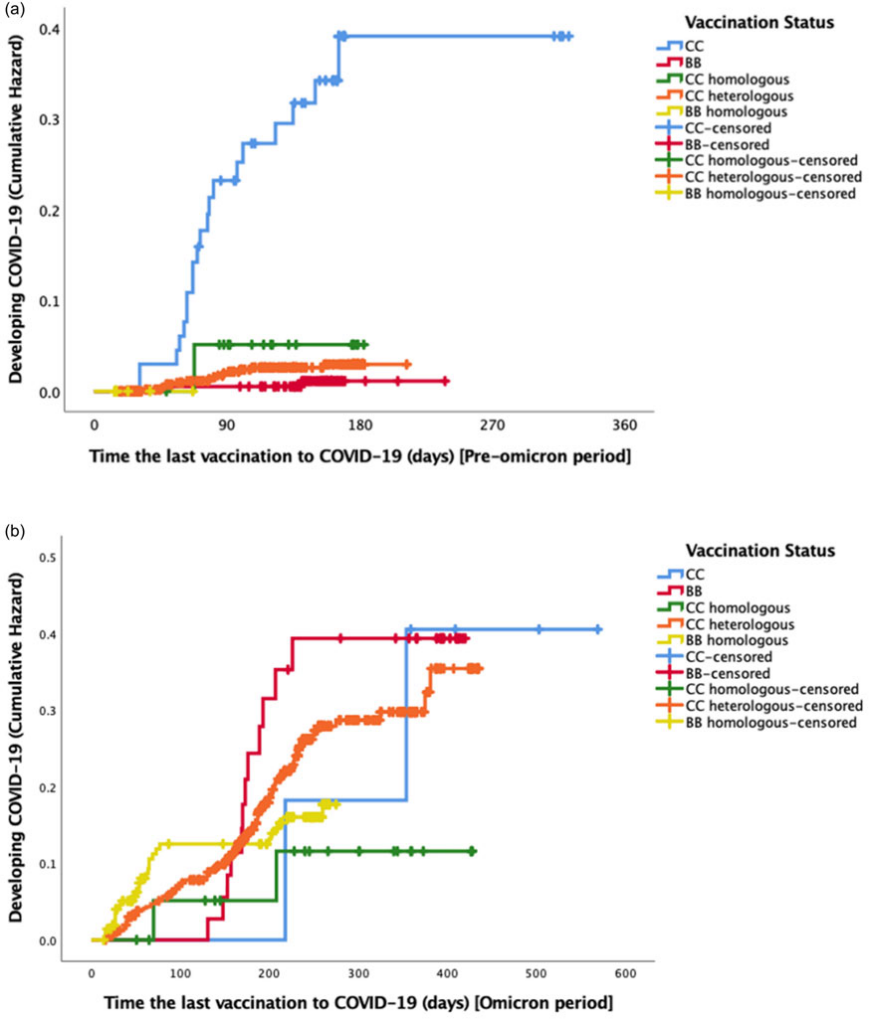
(a) Kaplan Meier curve for COVID-19 hazard for vaccine types during pre omicron period. (b) Kaplan Meier curve for COVID-19 hazard for vaccine types during Omicron period.

Cumulative hazard of symptomatic, PCR-confirmed COVID-19 infection over time was calculated with respect to COVID-19 vaccine schemes. In pre-Omicron period, those with a primary series of CoronaVac had the highest hazard rate for symptomatic COVID-19 (Figure 3a) (Log-rank test *P* value <.001); this association became non-significant after the Omicron variant became prominent in circulation (Figure 3b) (Log-rank test *p*-value = .231). Cox proportional hazards modeling for symptomatic, PCR-confirmed COVID-19 infection was used to further study protective effects of COVID-19 vaccines, adjusting for potential confounders. In the period preceding Omicron dominance, regardless of the primary series of COVID-19 vaccines, having at least one booster with Comirnaty was associated with a statistically significant reduction in COVID-19 hazard (Table 3): protective effect of Comirnaty continued after Omicron surge in the population, however, statistical significance was lost (Table 4). Table 4 presents that high compliance with non-pharmacologic preventive measures in social settings was statistically significantly protective against symptomatic COVID-19, regardless of the vaccination scheme.

**Table 3.** Symptomatic, PCR-confirmed COVID-19 infection hazard in Pre-Omicron Period (before January 1, 2022)

	Model 1 (n = 845)		Model 2 (n = 678)^a^		Model 3 (n = 639)^b^		Full Model (n = 678)^c^	
	HR (95% CI)	*p*-value	HR (95% CI)	*p*-value	HR (95% CI)	*p*-value	HR (95% CI)	*p*-value
CC or CC homologous	1.00	<.001	1.00	<.001	1.00	<.001	1.00	<.001
BB or BB homologous	.04 (.01–.16)	<.001	.04 (.01–.19)	<.001	.06 (.01–.37)	.002	.05 (.01–.36)	.003
CC heterologous	.10 (.05–.20)	<.001	.09 (.04–.19)	<.001	.07 (.03–.16)	<.001	.06 (.03–.14)	<.001
Age (years)	–	–	–	–	.58 (.40–.85)	.006	.49 (.32–.77)	.002
Grade IV	–	–	–	–	1.00	.279	1.00	.110
Grade V	–	–	–	–	2.13 (.59–7.66)	.248	2.78 (.63–12.34)	.179
Grade VI	–	–	–	–	3.37 (.76–14.92)	.110	5.79 (1.10–30.48)	.038

HR, hazard ratio. CI, confidence interval. ref., reference. B, Comirnaty. C, CoronaVac.

a Controlling for presence in crowded settings, masking and distancing in hospital, masking and distancing in social environment.

b Controlling for gender, faculty, comorbidity, current smoking status and body mass index.

c Controlling for gender, faculty, comorbidity, presence in crowded settings, masking and distancing in hospital, masking and distancing in social environment.

**Table 4.** Symptomatic, PCR-confirmed COVID-19 infection hazard in Omicron Period (January 1, 2022 and beyond)

	Model 1 (n = 853)		Model 2 (n = 689)^a^		Model 3 (n = 642)^b^		Full Model (n = 689)^c^	
	HR (95% CI)	*p*-value	HR (95% CI)	*p*-value	HR (95% CI)	*p*-value	HR (95% CI)	*p*-value
CC (ref.)	1.00	.246	1.00	.186	1.00	.709	1.00	.689
BB	1.12 (.25–4.98)	.886	.85 (.11–6.80)	.881	1.37 (.16–11.67)	.776	.80 (.09–6.84)	.838
CC homologous	.36 (.05–2.54)	.303	.41 (.04–4.61)	.470	.51 (.05–5.78)	.588	.38 (.03–4.46)	.439
BB homologous	.62 (.15–2.62)	.516	.46 (.06–3.43)	.445	.86 (.11–7.00)	.885	.47 (.06–3.83)	.482
CC heterologous	.90 (.22–3.63)	.877	.79 (.11–5.74)	.819	.79 (.11–5.93)	.821	.52 (.07–4.10)	.536
Masking/distancing in social life	–	–	.66 (.45–.95)	.027	–	–	.68 (.46–.99)	.042

HR, hazard ratio. CI, confidence interval. ref., reference. B, Comirnaty. C, CoronaVac.

a Controlling for presence in crowded settings, masking and distancing in hospital, masking and distancing in social environment.

b Controlling for age, gender, faculty, grade, comorbidity, current smoking status and body mass index.

c Controlling for age, gender, faculty, grade, comorbidity, presence in crowded settings, masking and distancing in hospital, masking and distancing in social environment.

P.S. Tables 3 and 4 differ in grouping of vaccine types given the scarcity of COVID-19 cases among vaccinated individuals before the Omicron surge, vaccine groups were merged as CC and CC homologous, BB and BB homologous, and CC heterologous.

## Discussion

The COVID-19 pandemic has been a major public health problem at global scale over the last 6 years. On May 4, 2023, the head of the World Health Organization (WHO) declared an end to COVID-19 as a “public health emergency of international concern” (PHEIC).^12^ The WHO’s declaration of “end of emergency status” should not be misleading, though. The Declaration stated a strong warning for continuing infection risk, underscoring the crucial need for surveillance. Even with full vaccine coverage, endurance of infection in societies could lead to emergence of new variants, which can threaten the effectiveness of available vaccines and even lead to new epidemic surges. Also, recent literature suggests up to 30% of long-term complications among COVID-19 patients, irrespective of severity of infections.^12,13^ Together, these reveal the public health importance of vaccination and underline the need for robust effectiveness studies.

Real-time comparative evaluations of available vaccines in different populations will enable valid and cost-effective policy decisions, provide concrete evidence on long-time safety concerns, besides illuminating vaccines’ immunogenic potential, regarding the interplay between cellular and humoral immunity in prevention of breakthrough infection. In this stage of the pandemic, national electronic health records (when available) could be the most efficacious investigation method for ambi-directionally studying effectiveness of various COVID-19 vaccines, together with the burden of adverse effects and long COVID-19 symptoms. Where countries do not yet have a specific surveillance for COVID-19, prospective cohorts seem to be the best method for real-life effect estimations of COVID-19 vaccines.

Our cohort was unique in many aspects.^6^ The study was initiated by volunteer faculty members as a social responsibility project, besides its expected scientific assets, to reach our health science students for support their well-being over the pandemic; enabling early diagnosis, isolation, and treatment, as needed. Emergency Use Authorization (EUA) for CoronaVac in Türkiye was based on unplanned interim analysis of local phase 3 data (with 29 cases) and interim findings of a relatively larger phase 3 trial in Brazilian population.^14^ Thus, a prospective cohort of 1,133 fully vaccinated students, with at least one completed visit, was very informative for robust effectiveness estimations. One-center experience limited external validity of our incidence rates; yet, intrinsic validity was maximized by means of standardized inquiries, robust lab testing, long follow-up times, and adjustments for various individual and behavioral characteristics. Follow-up of 478 466 person-days is a major strength of our study compared to earlier CoronaVac studies.^15–17^

Our main finding of interest was higher protective capacity of Comirnaty compared to CoronaVac, controlling for potential risk factors. Consistent with literature, Comirnaty boosters increased protective ability of primary series with CC.^15^

Confirming earlier work, administration of a Comirnaty dose/booster was also associated with a significant increase in neutralizing antibody titers, which stayed significant adjusting for potential confounders.^5,18–20^

Kaplan Meier analysis of case status by antibody titers, controlling for the time (days) between the most recent antibody measurement and time to case/non-case status. We used a start-stop approach for all participants for 1737 valid periods; time-to-event was calculated from the most recent antibody measurement to case status/censoring time. Inclusion of the same individual (for up to 3 periods) in survival analysis might have decreased variance yet enabled us to study antibody-disease associations for 3-month periods in parallel to variations in circulating variants in Türkiye. Vaccine-related antibodies were significantly higher with Comirnaty compared to CoronaVac and stayed at high levels for significantly longer periods. A similar, significantly higher protection from symptomatic COVID-19 cases was associated with Comirnaty use in pre-Omicron period but lost significance after the Omicron surge. This finding was contradicting with ample evidence suggesting higher effectiveness of Comirnaty against CoronaVac, despite its decreased potency against Omicron variant.^19–22^ We tried to explain this through detailed assessment of antibody levels over time to check whether undetected breakthrough infections could intervene with vaccine-induced protection.

The prominent antibody re-surge 90–180 days after valid vaccination was remarkable and varied disproportionally by the type of COVID-19 vaccine used. Time of re-surge in antibody levels appeared earlier among CoronaVac recipients (around 90 d), regardless of primary series and/or booster doses. Unfortunately, our repeated antibody measures were limited in the cohort (ie, 546 students with 2 or more antibody measures). Individual-based trends in anti-SARS-COV-2 spike RBP IgG antibodies were not sufficiently informative, and potential confounders for individual antibody response might have biased the trends. With this in mind, we hypothesized that the apparent increase in antibody levels starting from 90 days after vaccination were due to natural, undetected (mostly asymptomatic or mild) COVID-19 infections in young adults. Supporting this hypothesis, breakpoints in trends (ie, beyond 90 and 180 d, respectively for C and B) were compatible with expected decrease in antibody levels below sufficiently protective levels, making individuals prone to infections.^23^ Our finding reveals that undetected infections in vaccine studies (including clinical trials for seasonal vaccines) could bias the comparative effectiveness of COVID-19 vaccines, favoring the effect size of vaccine with low durability. This finding also supported our finding of no significant difference across vaccine effects in the Omicron period.

Unexpected antibody level upsurges among vaccinated individuals, in absence of any new vaccination or diagnosed COVID-19 infection might indicate breakthrough COVID-19 infections as vaccine-induced immunity decreases, when the need for testing is ignored. Immunity evoked by the breakthrough infection will add onto vaccine-induced anti-SARS CoV-2 antibody levels and would have a synergic effect against symptomatic infection. Considering breakthrough infection-induced immunity as an intermediate factor (mediator) in investigating vaccine effectiveness against symptomatic infection could mislead researchers on “direct” protective effects of vaccine types. Methodologically, adjustment is not a default for mediators’ effect, if an overall effect of the exposure is of concern. In this study, however, those in the vaccine arm with less effectiveness were more likely to face an earlier and more profound (asymptomatic) infection, leading to a higher immunity/protection against future symptomatic COVID-19 cases “Differential” incidence of asymptomatic infections (as an intermediate variable) might lead to a bias toward the null in comparing vaccines’ effectiveness. That is, relatively higher effectiveness of one vaccine might be obscured, if mediator’s role is not analyzed carefully.

The main limitation of our study was the inability to integrate periodic PCR testing into follow-up and to depend on self-evaluation of students for presence of any COVID-19-like symptom. We highly recommend future researchers to conduct periodic PCR testing for participants to improve the epidemiologic quality of vaccine-effectiveness studies. Repeated PCR testing with concurrent antibody testing will be clearly warranted to reveal the relationship between antibody levels and disease status, besides supporting our hypothesis of unexpected antibody re-surges months after vaccination due to undetected COVID-19 cases.

Comparative analysis of effectiveness of vaccines in real life is a methodologically complex issue; besides antibody levels, we need to adjust for numerous confounders. We used different models to control for major confounders, yet the potential for residual confounding cannot be excluded. Reasons for and intentions to select one type of COVID-19 vaccine from available choices could be a strong confounder by itself, yet, in our study vaccine type was mainly determined by the availability of vaccine, rather than an individual choice for the primary series. Behavioral factors, prevalence of infection in the population etc. will all modify the attributable risk; e.g, we studied the effect of masking. Masking was an independent predictor of protection in the Omicron period, but not earlier. Self-reporting and recall might have biased the information on (proper) mask use, yet obligatory use of masking early in the pandemic could have hidden masking’s effect in the pre-Omicron period. Lastly, the cohort design limits generalizability of the findings.

In conclusion, our findings are consistent with literature regarding higher antibody levels and protective ability of Comirnaty versus CoronaVac; confirming protective value of both vaccines in a young, high risk group.^24–26^ Our results are novel to underscore that focusing solely on symptomatic infections may misguide assessment of vaccine effectiveness in populations where asymptomatic infection burden is not monitored.^27^

## Supporting information

10.1017/ash.2026.10338.sm001 Cakir et al. supplementary material 1 Cakir et al. supplementary material

10.1017/ash.2026.10338.sm002 Cakir et al. supplementary material 2 Cakir et al. supplementary material

## Data Availability

The data underlying this article will be shared on reasonable request to the corresponding author.

## Supplementary material

The supplementary material for this article can be found at https://doi.org/10.1017/ash.2026.10338.
