# Supplementary material for: Comparative effectiveness of COVID-19 vaccines among health students, focusing on methodological concerns
Source: Antimicrob Steward Healthc Epidemiol. 2026 Apr 7;6(1):e91. doi: 10.1017/ash.2026.10338 (PMC13104580; doi:10.1017/ash.2026.10338)
Supplement: Cakir et al. supplementary material 1 — Cakir et al. supplementary material [file S2732494X26103386sup001.docx]

**Supplementary Table 1.** Vaccination Characteristics of Study Participants as of September 9, 2022.

| **Vaccination Status (as of September 9, 2022)** | **n** | **%** |
| --- | --- | --- |
| Incomplete primary vaccination^a^ | 3 | 0.3 |
| CoronaVac-CoronaVac (primary scheme) | 61 | 5.4 |
| Comirnaty-Comirnaty (primary scheme) | 39 | 3.4 |
| CoronaVac-homologous^b^ | 26 | 2.3 |
| CoronaVac-heterologous^c^ | 778 | 69.2 |
| Comirnaty-homologous^d^ | 213 | 19.0 |
| Comirnaty-heterologous^e^ | 1 | 0.1 |
| Miscellaneous^f^ | 3 | 0.3 |
| **COVID-19 positivity (as of September 9, 2022)^g^** | 203 | 17.9 |

^a^ Single dose Comirnaty or CoronaVac

^b^ Triple or quadruple dose CoronaVac

^c^ CoronaVac + CoronaVac + single, double or triple dose (s) of Comirnaty, as booster

^d^ Comirnaty + Comirnaty + single or double dose(s) of Comirnaty, as booster

^e^ Comirnaty + Comirnaty + Turkovac, as booster

^f^ CoronaVac + single, double or triple dose(s) of Comirnaty

^g^ Symptomatic COVID-19 case, with a PCR confirmation

**Supplementary Table 2.** The most recent antibody titers and relevant follow-up times

| **Variable** | **Mean ± Std.Dev.^a^** | **Median (25%-75%)** | |
| --- | --- | --- | --- |
| Anti-SARS-COV-2 spike- RBP lgG (BAU/mL)^a^ (n=1119) | 1729.3±1780.1 | 1126.4 (165.1-2677.8) | |
| Time from the last valid vaccination through the most recent antibody testing (days)^b^ (n=1121) | 110.4±60.7 | 113.0 (81.0-126.0) | |
| Time since the most recent vaccine, stratified on the Type of Circulating Variant^c^ | | | |
| Pre-omicron period (n=1117) | 114.6±63.5 | | 135.0 (35.0-171.0) |
| Omicron period (n=854) | 216.5±102.8 | | 225.0 (162.8-265.3) |

^a^ RBP: Receptor binding protein, BAU: Binding antibody unit [7]

^b^As used in linear regression models. Started at the 14^th^ days after at least 2 doses of (or booster) COVID-19 vaccination.

^c^ As used in Kaplan Meier curves and for denominators of incidence rate calculations. Started 14^th^ days after the most recent valid COVID-19 vaccination through COVID-19-positivity (cases) or for non-cases, the last day when COVID-19 information was available (i.e., censoring date).

**Supplementary Table 3.** Multiple Linear Regression Model for anti-SARS-COV-2 spike- RBP IgG Antibody levels (in BAU/ml)

| **Variables in the Model** | **Standardized ß** | **ß** | **95% Confidence interval of ß** | **p-value** |
| --- | --- | --- | --- | --- |
| Male gender | 0.11 | 402.33 | 191.69 – 612.98 | <0.001 |
| Faculty of Medicine | -0.19 | -978.36 | -1,279.52 – -677.21 | <0.001 |
| Time from vaccination through antibody testing (days) ^a^ | | | | |
| < 90 days (reference) |  |  |  |  |
| 90 – 179 days | -0.22 | -793.13 | -1,061.68 – -524.59 | <0.001 |
| ≥ 180 days | -0.10 | -666.95 | -1,072.07 – -261.84 | 0.001 |
| Vaccination number/type^b^ | | | | |
| CC (reference) |  |  |  |  |
| BB | 0.04 | 232.54 | -202.70 –667.77 | 0.300 |
| BB with homologous booster(s) | 0.37 | 2,069.08 | 1,674.13 – 2,464.03 | <0.001 |
| CC with homologous booster(s) | 0.002 | 29.40 | -828.90 – 887.70 | 0.950 |
| CC with heterologous booster(s) | 0.41 | 1,470.41 | 1,168.34 – 1,772.49 | <0.001 |

^a^If the most recent vaccination was within 14 days from the antibody testing, we considered the preceeding vaccination status, as valid for vaccine-related antibody levels.

^b^ B: Comirnaty, C: CoronaVac

Adjusted for age, adherence to non-pharmacological measures in hospital or public places, mingling with the crowds, history of prior symptomatic, PCR-confirmed COVID-19 and comorbidity. Model R^2^= 0.29
