# Supplementary figures and images for: Comparative effectiveness of COVID-19 vaccines among health students, focusing on methodological concerns
Source: Antimicrob Steward Healthc Epidemiol. 2026 Apr 7;6(1):e91. doi: 10.1017/ash.2026.10338 (PMC13104580; doi:10.1017/ash.2026.10338)

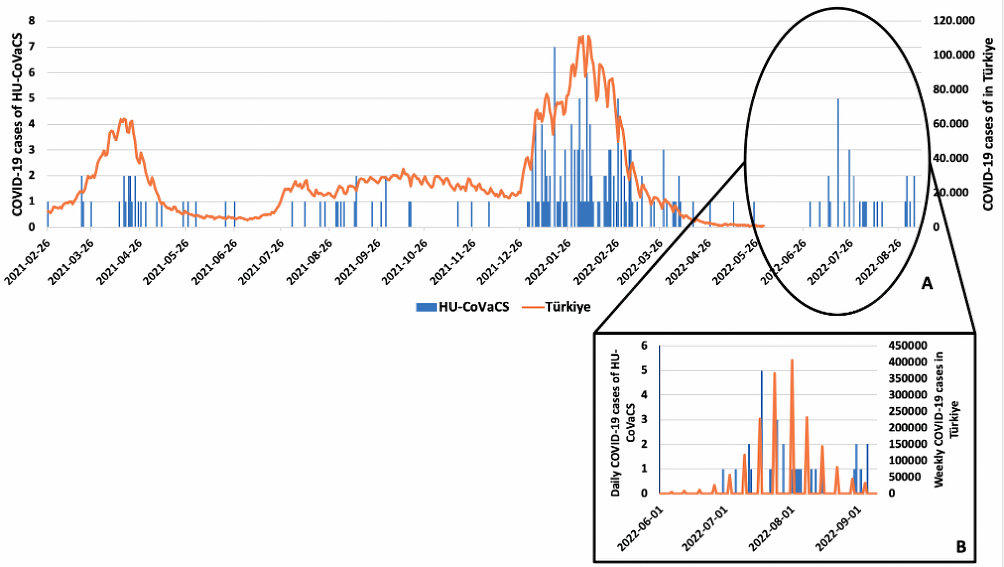

Supplement: Cakir et al. supplementary material 2 — Cakir et al. supplementary material [file S2732494X26103386sup002.tiff]
